# Electrochemical Reduction of O_2_ in Ca^2+^‐Containing DMSO: Role of Roughness and Single Crystal Structure

**DOI:** 10.1002/cssc.202100364

**Published:** 2021-05-13

**Authors:** Andreas Köllisch‐Mirbach, Inhee Park, Martina Hegemann, Elke Thome, Helmut Baltruschat

**Affiliations:** ^1^ Institut für Physikalische und Theoretische Chemie Universität Bonn Römerstraße 164 53117 Bonn Germany

**Keywords:** batteries, calcium, crystal structures, electrochemistry, oxygen reduction

## Abstract

In this study, the oxygen reduction reaction (ORR) in Ca^2+^‐containing dimethyl sulfoxide (DMSO) at well‐ordered and rough electrode surfaces is compared by using cyclic voltammetry, differential electrochemical mass spectrometry, rotating ring disk electrode, and atomic force microscopy measurements. Slightly soluble CaO_2_ is the main product during early ORR on gold electrodes; after completion of a monolayer of CaO and/or CaO_2_, which is formed in parallel and in competition to the peroxide, only superoxide is formed. When the monolayer is completely closed on smooth annealed Au, no further reduction occurs, whereas on rough Au a defect‐rich layer allows for continuous formation of superoxide. CaO_2_ formed either via two subsequent 1 $e^{-}$ transfer steps or by disproportionation of superoxide may be deposited on top of the CaO/CaO_2_ adsorbate layer. The slow dissolution of the peroxide particles is demonstrated by AFM. Whereas a smooth CaO/CaO_2_‐covered electrode shows severe deactivation and a CaO/CaO_2_‐covered rough electrode allows for diffusion‐limited superoxide formation, on single crystals peroxide formation is more pronounced. The reason is most likely the lack of nucleation sites for the blocking CaO/CaO_2_ layer. RRDE investigations showed sluggish reoxidation kinetics of the dissolved peroxide, which are most likely due to ion pairing with Ca^2+^. The apparent transfer coefficient is estimated by using variation of the electrode roughness, confirming the result of the usual Tafel analysis and indicating an equilibrated first 1 $e^{-}$ transfer.

## Graphical abstract

**Rough with the smooth**: This work highlights the role of adsorbate formation and of deposited CaO_2_ during oxygen reduction in Ca^2+^‐containing dimethyl sulfoxide. By systematically varying the electrode roughness, the reaction mechanism and product distribution and the crucial influence of electrode structure thereupon are elucidated.

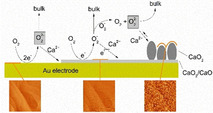

## Introduction

Increasing necessity for renewable energy sources requires means to store the energy, as it cannot be easily generated on demand. Not only pumped‐storage power plants and energy storage by electrochemical generation of hydrogen, but also the storage in large scale batteries is a possibility one could think of looking towards the future. As next‐generation batteries, metal‐air batteries might be the way to go in the future. Their big advantage is not only the high energy density, which is primarily important for electromotive applications, but also – and probably more importantly – the fact that less abundant elements like Co are not needed in stoichiometric amounts for insertion materials at the cathode. Though metal‐air battery research is a common research topic nowadays, the performance of this type of battery still remains insufficient and a lot of work still needs to be done. Li‐O_2_,[^1^, ^2^, ^3^, ^4^, ^5^, ^6^, ^7^, ^8^] Na‐O_2_,[^9^, ^10^, ^11^, ^12^] and Mg‐O_2_[^13^, ^14^, ^15^] systems already were topic of research and looking towards further cations like potassium[^16^, ^17^, ^18^] and calcium seems reasonable, as the ORR product distribution depends on the cation.[^4^, ^19^, ^20^, ^21^, ^22^] The use of more abundant active materials than lithium for the anode may also be necessary for a wider application. Calcium was already proposed as interesting candidate, as it is one of the most abundant elements in the earth's crust and still has a sufficiently high theoretical energy density (Ca‐air) to replace Li‐ion batteries. Both ORR/OER[^21^, ^22^] and metal deposition[^23^, ^24^] were investigated in Ca‐ion containing aprotic electrolytes.

The ORR and OER were investigated on several cathode materials. For the ORR in Ca(ClO_4_)_2_ containing DMSO on Pt, Rh, Glassy Carbon and Ru a one electron process was observed, while for Au a two electron process is found.^[21]^ It was shown, that Au plays a special role in the mechanism, as on gold in Mg^2+^, Ca^2+^, Sr^2+^ and Ba^2+^ containing DMSO, the two electron process is dominating the ORR, which is not the case for Glassy Carbon and platinum electrodes.^[22]^ This is of special interest, as the formation of $O_{2}^{-}$ and further solution mediated disproportionation to $O_{2}^{2-}$ and $O_{2}$ might lead to significant amounts of highly reactive singlet oxygen, which will decompose the electrolyte.[^25^, ^26^, ^27^] Upon reducing the relative amount of generated $O_{2}^{-}$ , one might increase the reversibility and thus the cycle life of the battery. At this point, the current work starts with further investigation on the ORR and OER in Ca(ClO_4_)_2_ containing DMSO using techniques like DEMS to identify oxygen consumption/evolution and RRDE to further investigate the nature of the generated oxygen species during the ORR. The influence of the electrode surface structure was taken into account by using single crystalline electrodes on the one hand and rough electrodes on the other. A similar approach was also used to investigate the ORR/OER in case of Li^+^ containing DMSO in our earlier work.^[28]^ We found that mostly Li_2_O_2_ is formed on rough electrodes during the ORR and thus showed once again, that the surface structure strongly affects the electrochemical reactions. This work strongly focuses on the ORR/OER mechanism in Ca^2+^‐containing DMSO and the influence of the surface structure (single crystal orientation on the one hand and high roughness factors on the other). It supplements a previous study in which already a transition from mixed $O_{2}^{-}$ and $O_{2}^{2-}$ formation to exclusively $O_{2}^{-}$ formation was observed on smooth electrodes.^[29]^ The study further showed that the blocking thin film mainly consist of Ca(O_2_)_2_, CaO_2_ and CaO (identified by XPS) and is formed on Au and Pt electrodes. In other previous studies we have shown that reliable and clean double layer studies can be performed on Au(111) in aprotic solvents (e. g., pzc measurements).^[30]^

## Experimental Section

### Electrodes

The gold electrodes used in this study had a diameter of 1 cm resulting in a geometrical surface area of 0.785 cm^2^, while RRDE disk electrodes had a diameter of 0.5 cm and 0.457 cm and thus a geometrical surface area of 0.196 cm^2^ (exchange disk electrode) or 0.164 cm^2^ (thin gap disk electrode) respectively. Ring electrodes were also made of gold, while the theoretical collection efficiency ($N_{0}$ ) was 0.25 for the exchange disk electrode and 0.22 for the thin gap disk electrode.

### RRDE setup

The RRDE experiments were conducted in a usual H‐Cell under argon atmosphere. The Au working electrode (WE), Au counter electrode (CE) and Ag reference electrode (RE) were connected to a bipotentiostat (TACUSSEL BIPAD B1), while electrode rotator was connected to a PINE rotation control. Counter electrode was a gold sheet and the reference electrode was a silver wire immersed into 0.1 m AgNO_3_ containing DMSO. Disc currents were normalized to the geometrical surface area of the disk electrode ($A_{geo}^{disk}$ ); ring currents were normalized not only to the collection efficiency$\;N_{0}$ but also to the geometrical surface area of the disk electrode ($A_{geo}^{disk}$ ) for 1 to 1 comparability.

### DEMS setup

DEMS measurements were conducted using a dual thin‐layer flow‐through cell, where WE, CE and RE were connected to a homebuilt bipotentiostat. The electrolyte outlet was connected to a peristaltic pump (SPETEL Perimax). The CEs were gold wires and the RE was a silver wire immersed into 0.1 m AgNO_3_ in DMSO. The RE was contacted to the working electrolyte via a Teflon tube (sealed with a rough glass bead) filled with the silver containing solution. The end of the glass bead was immersed into the working electrolyte, while the open end was immersed into the RE electrolyte. The size of the thin‐layer compartment of the WE and of the compartment of the porous Teflon membrane (serving as the interface to the vacuum) were defined by a Teflon spacer of 200 μm and 100 μm thickness and a diameter of 6 mm (electrode area of 0.283 cm^2^), as in our previous study.^[28]^ More detailed information about the DEMS setup and the dual thin‐layer cell can be found elsewhere.[^22^, ^31^, ^32^, ^33^, ^34^] The faradaic current in DEMS measurements was smoothed using the Savitzky‐Golay method (100 mV window) to account for the periodic signal caused by the peristaltic pump.

### AFM setup

A disc‐type Au(111) single crystal (diameter: 10 mm; thickness: 3 mm) purchased from MaTecK GmbH was used as working electrode. Gold and platinum wires were used as counter and quasi‐reference (Pt/PtO) electrodes, respectively. The standard sealed chamber (Agilent 5500) was purged with Ar before transferring the AFM cell. After the AFM‐cell filled with electrolyte was loaded on the AFM, Ar was purged for around 10 min because the AFM‐cell was exposed to the atmosphere during assembling and transfer. O_2_ was purged during the experiment to maintain the concentration of oxygen in the electrolyte (100 % oxygen saturation). Sharp silicon tips (PPP‐CONTSC, tip radius <10 nm) were used with normal spring constants of 0.1±0.05 N/m. For AFM measurements the single crystals were prepared by flame annealing.

### Electrode cleaning

Prior to any experiment, the electrodes were cycled in 0.5 m H_2_SO_4_ until the well‐known shape of the cyclic voltammogram (CV) could be observed. In case of contaminations, the electrodes were cleaned further. The gold electrodes were oxidized in 0.5 M H_2_SO_4_ until the surface was covered by gold oxide. Then the formed gold oxide was removed in concentrated hydrochloric acid. After the cleaning procedure, the voltammetry was checked again to exclude remained contamination.[^35^, ^36^]

### Preparation of single crystals

The single crystals were cleaned as described above and annealed in a N_2_‐purged induction heat cell afterwards. After the annealing, the crystals cooled down in the same cell above N_2_‐saturated Milli‐Q water.[^35^, ^36^] Upon reaching room temperature, the electrodes were characterized in H_2_SO_4_ once every day prior to an experiment to proof the setup. When the setup showed good performance, the electrodes were again prepared and transferred to the experimental setup.

The electrode roughening and the single crystal preparation procedures were the same as in an earlier publication.^[28]^ The electrode roughness of polycrystalline electrodes equals one unless stated otherwise. The single crystals used for AFM experiments were prepared by flame annealing.

### Chemicals

Calcium perchlorate tetrahydrate (99 %, Sigma Aldrich) was dried (8 h) under reduced pressure to obtain a white powder of anhydrous Ca(ClO_4_)_2_. Tetrabutylammonium perchlorate (≥99 %, Sigma Aldrich), silver nitrate (99 %, CHEMPUR), and Dimethyl sulfoxide (99.7 %, extra dry, over molecular sieve) were used as received. DMSO, CaClO_4_ and TBAClO_4_ were stored inside an *MBraun* glovebox under argon atmosphere. For purging and electrolyte saturation argon (99.999 %, Air liquide), nitrogen (99.999 %, Air liquide), oxygen (99.9995 %, Air liquide) and argon oxygen mixture (80 %/20 %, Air liquide) were used.

## Results

### Investigations in resting/quiescent solution

We already reported that the oxygen reduction reaction (ORR) in Ca^2+^‐containing DMSO on Pt, Rh, Glassy Carbon and Ru leads to a large extend to the formation of soluble Ca(O_2_)_2_ whereas on gold electrodes, the oxygen reduction reaction leads to the formation of soluble CaO_2_.^[21]^ To further understand the effect of the electrode and the electrode surface structure concerning the ORR, experiments on gold single and poly crystals were conducted.

**Figure 1:**
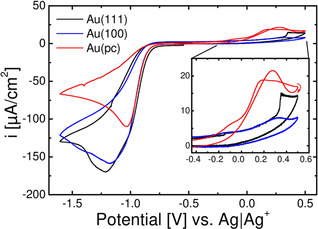
The first recorded cyclic voltammogram for single crystalline and polycrystalline gold electrodes at 10 mV s^−1^ in (20 % O_2_+80 % Ar)‐saturated 0.1 m Ca(ClO_4_)_2_ containing DMSO after immersion at −0.5 V vs Ag/Ag^+^. The current is normalized to the geometrical surface area. Inset shows OER region more detailed. The polycrystalline electrode was prepared/annealed like the single crystals. Only the 1^st^ sweep is shown because of probable changes in the surface structure in subsequent scans (see the Supporting Information).

Figure 1 shows the first cyclic voltammogram of single crystalline and polycrystalline gold electrodes in (20 % O_2_+80 % Ar)‐saturated 0.1 m Ca(ClO_4_)_2_ containing DMSO vs Ag/Ag^+^ at 10 mV s^−1^ after immersion at −0.5 V. Starting in cathodic direction, the absolute value of the current density increases to a maximum close to −1.0 V for the polycrystalline gold electrode. In case of Au(111) the feature at −1.0 V appears as a shoulder prior to a peak close to −1.25 V. In case of Au(100) no such feature close to −1.0 V is visible as the current increases to a maximum close to −1.15 V. After the maxima the current decreases, while in case of Au(111) and Au(100) a shoulder is visible in the decreasing current. Furthermore, the ORR onset in case of Au(111) is slightly shifted positive compared to Au(100) and Au(pc), which almost overlap during the ORR onset. After going to zero in anodic direction, the oxidation starts with a shoulder at −0.14 V, followed by a broad peak of low current in case of Au(100), a sharp peak of slightly larger current in case of Au(111) and a broad peak with a much larger current in case of Au(pc). After the change in sweep direction at 0.5 V, the current decays monotonously to zero in case of Au(111) and Au(100). In case of Au(pc) the current increases again to a maximum close to 0.25 V before it decreases to zero.

Calculating the theoretical ORR peak current density for this system with the Randles–Sevcik equation for reversible (or totally irreversible) processes (See the Supporting Information) leads to 153.5 μA cm^−2^ (or 85.5 μA cm^−2^ for $z_{\alpha }$ =1, 120.9 μA cm^−2^ for $z_{\alpha }$ =2) for a two electron process and for a one electron process 54.5 μA cm^−2^ (or 42.7 μA cm^−2^ for $z_{\alpha }$ =1), where $z_{\alpha }$ is the number of electrons transferred in the rds. The comparison with the experimental values which are larger than 100 μA cm^−2^ indicates peroxide as main product. Combining this fact with the lower number of defects in single crystalline surfaces compared to poly crystalline surfaces, explains the observed differences in ORR and OER current: Thus, the nucleation of CaO_2_ (or possibly CaO) is hindered at both Au(111) and Au(100) surfaces but facile at Au(pc) surfaces. This leads to a faster decay in ORR current at Au(pc) electrodes and a larger OER current, owing to increased deposition of CaO_2_ on the electrode surface as compared to Au(111) and Au(100). This also explains the much higher oxidation charge of ca. 1860 μC cm^−2^ at the polycrystalline surface as compared to the single crystal surfaces.

### Atomic force microscopy (AFM) investigations

**Figure 2:**
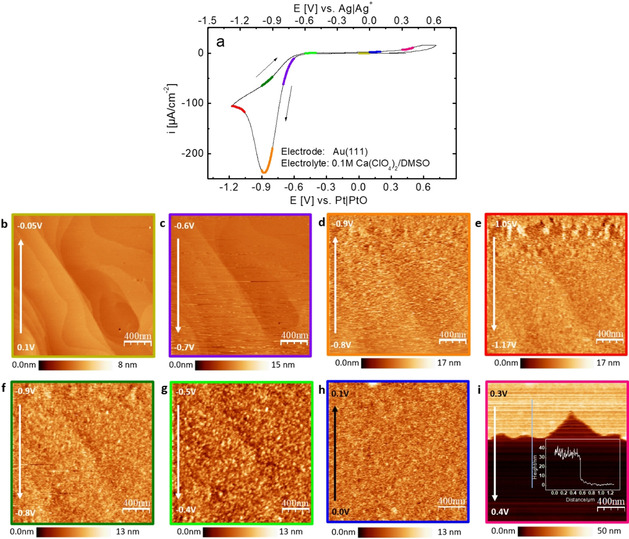
a) Voltammetry of Au(111) in an AFM‐cell at 5 mV s^−1^ in 0.1 m Ca(ClO_4_)_2_ containing DMSO (100 % oxygen saturated). b–i) In situ AFM images show the topography on Au(111) in the indicated potential ranges upon discharge (b–e) and on charging (f–i); all potentials refer to Ag/Ag^+^. The inset in (i) shows the height profile. The color code (AFM‐frame and CV‐line) denotes the actual potential region. The arrow in the AFM images indicate the scan direction. Integral and proportional gains were 6 and 7, respectively. Scan size was 2×2 μm^2^ and the scan rate was 19 nm s^−1^.

To further investigate the adsorbate/deposit an in situ AFM measurement was conducted in 0.1 m Ca(ClO_4_)_2_ containing DMSO on Au(111). Figure 2a shows the CV in the AFM cell.

Figure 2b shows that prior to ORR large and clean terraces can be observed between 0.0 V and −0.15 V vs. Ag/Ag^+^. When the potential passed the onset of ORR, some particles are deposited on the surface appearing as noise in the Figure 2c and these particles grow to a dense layer upon sweeping more negative than −0.8 V vs. Ag/Ag^+^ (Figure 2d,e). Then continuing in anodic direction after the negative potential limit of −1.27 V vs. Ag/Ag^+^, the particles seem to grow continuously until a potential of −0.5 V vs. Ag/Ag^+^ is attained. The apparent growth of the particles during the anodic scan indicates a further deposition of solid CaO_2_ (that this is really CaO_2_ will be shown below) from $O_{2}^{2-}$ directly formed at the electrode or formed via disproportionation of the dissolved superoxide (formed at the most negative potentials). Ostwald ripening might also play a role. While sweeping the potential from −0.5 V to −0.1 V vs. Ag/Ag^+^, the interaction between the AFM tip and the electrode surface became unstable and the tip was retracted. The subsequent images therefore were recorded at a different position. Figure 2h was recorded after re‐approaching the tip. It is remarkable that the size of the particles is reduced at a more positive potential than −0.5 V vs. Ag/Ag^+^. We assume that the dissolution of peroxide leads to the reduced size, but this may also be caused by the shift in the scanned area. Figure 2i shows that the deposited particles seem to quickly dissolve at the potential of OER without any appreciable charge flow and the inset shows that the height of the deposited layer is around 30 nm. Furthermore, in a similar experiment (Figure S1), the potential was stopped at −0.65 V vs. Ag/Ag^+^ (ca. −0.55 V vs. Pt/PtO) (after ORR) and images were taken every 3 min. After 6 min, the particles formed during ORR were gone during the scan. This observation further demonstrates the slow dissolution of the formed particles without oxidation and also explains that hardly any charge is flowing during the dissolution in Figure 2i. Note that the dissolution of the particles might have been accelerated by the scanning tip (e. g., by induced convection by moving AFM tip). This measurement clarified that a bulk layer of CaO_2_ forms at the electrode in the absence of convection, but slowly dissolves without oxidation. However, at this point it is not yet clear whether the thick layer is the reason for the blockade of the electrode.

### Investigations under flow‐through conditions using differential electrochemical mass spectrometry

Differential electrochemical mass spectrometry (DEMS) provides information about the product distribution, the consumption and generation of volatile species and furthermore the number of transferred electrons during ORR and OER after a calibration measurement.

**Figure 3:**
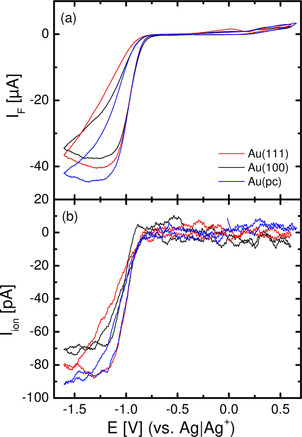
a) Cyclic voltammetry of single‐ and polycrystalline gold electrodes in (20 % O_2_+80 % Ar)‐saturated 0.4 m Ca(ClO_4_)_2_ containing DMSO at 10 mV s^−1^) and 5 μl s^−1^ flow rate using a thin‐layer flow‐through cell. b) Potential‐dependent ion current for mass 32 (O_2_) for single‐ and polycrystalline gold electrodes.

Figure 3a shows the cyclic voltammetry (second cycle) of polycrystalline and single crystalline Au electrodes in 0.4 m Ca(ClO_4_)_2_ containing DMSO saturated with (20 % O_2_+80 % Ar), together with the ion current for mass 32 (O_2_; Figure 3b).

**Figure 4:**
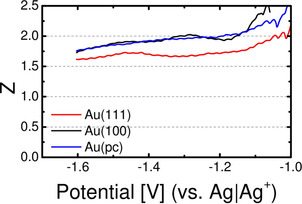
Potential‐dependent number of transferred electrons per O_2_ during the ORR in cathodic direction for single‐ and polycrystalline gold electrodes in (20 % O_2_+80 % Ar)‐saturated 0.4 m Ca(ClO_4_)_2_ containing DMSO at 10 mV s^−1^ and 5 μl s^−1^ flow rate using a thin‐layer flow‐through cell.

The absolute faradaic current increases to a plateau for the diffusion‐limited formation of predominantly CaO_2_, indicated by the number of transferred electrons (Figure 4). The number of transferred electrons starts from *Z*≈2 (in case of Au(111) *Z*≈1.7) at −1.1 V and stays constant during the remaining cathodic sweep. This in combination with the fact, that almost no reoxidation current is observed, leads to the assumption, that predominantly soluble CaO_2_ is formed during the ORR on all gold electrodes, as already suggested in Ref. [21]. No strong effect of the single crystal surface structure is observed (only in case of Au(111), the number of transferred electrons during ORR is slightly lower compared to Au(100)/Au(pc); this effect might be due to a slight change in calibration constant of the spectrometer).

Comparing the ORR during cathodic and anodic sweep one observes a hysteresis, indicating a change in electrode activity, whereas for polycrystalline gold electrodes the hysteresis is smaller. This is plausible, as we assume the formation of an insoluble (adsorbed) CaO_2_/CaO layer on the electrode surface (this will be discussed in detail in the RRDE chapter below). This hysteresis was already found when comparing gold with Glassy Carbon in Ca^2+^‐containing DMSO.^[21]^ As already observed in Figure 1, also under the conditions of Figure 3 the peroxide reoxidation is not finished at the end of the anodic scan, but continues after potential reversal.

**Figure 5:**
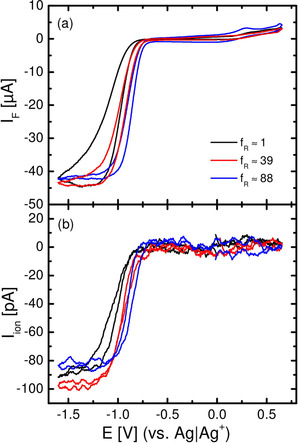
a) Cyclic voltammetry of roughened polycrystalline gold electrodes in (20 % O_2_+80 % Ar)‐saturated 0.4 m Ca(ClO_4_)_2_ containing DMSO at 10 mV s^−1^ and 5 μl s^−1^ flow rate vs Ag/Ag^+^ using a thin‐layer flow‐through cell. b) Potential‐dependent ion current for mass 32 (O_2_).

The effect of electrode roughness is shown in Figure 5. Overall, the cyclic voltammetry for Au does not change much with increasing roughness. The hysteresis is reduced, indicating less blocking for rougher electrodes. In the OER region, an oxidation peak only is clearly visible for the highest roughness factor. For all electrode roughness factors, the maximum absolute (limiting) current during the ORR remains the same, indicating that the CaO_2_ formation really is diffusion limited. The half‐wave potential of the ORR shifts positively with increasing electrode roughness, owing to the increasing effective rate constant; this will be further analyzed below.

**Figure 6:**
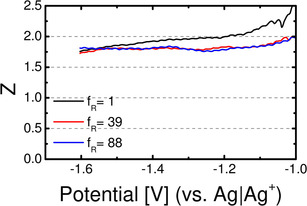
Potential‐dependent number of transferred electrons per O_2_ during the ORR in cathodic direction for roughened gold electrodes in (20 % O_2_+80 % Ar)‐saturated 0.4 m Ca(ClO_4_)_2_ containing DMSO at 10 mV s^−1^ and 5 μL s^−1^ flow rate using a thin‐layer flow‐through cell.

Figure 6 shows the potential‐dependent number of transferred electrons for the roughened gold electrodes. The number of transferred electrons constantly stays above a value of 1.7, indicating that the two‐electron process is predominant during the whole ORR on gold surfaces.

### Rotating ring disk electrode (RRDE) investigations

**Figure 7:**
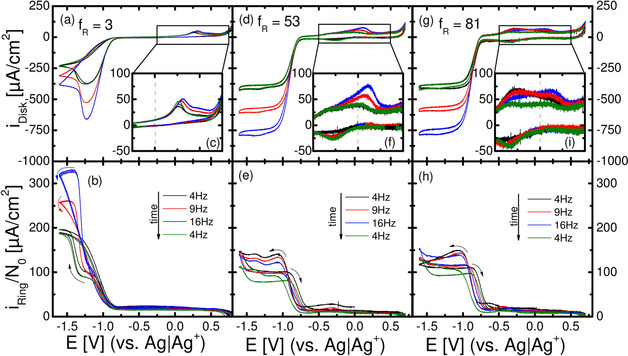
Cyclic voltammetry of roughened gold electrodes (exchange disk electrodes) in (20 % O_2_+80 % Ar)‐saturated 0.1 m Ca(ClO_4_)_2_ containing DMSO at 20 mV s^−1^, E_ring_=0.4 V and 4–16 Hz rotation frequency vs Ag/Ag^+^ using a usual H‐cell. Disk current density is shown in (a), (d), and (g). The ring current (b,e,h) is normalized to the collection efficiency $N_{0}$ and $A_{geo}^{disk}$ . The anodic signal is magnified in (c), (f), and (i), and the dashed grey line denotes 0 V.

To demonstrate the effect of higher convection and different roughness factors, Figure 7 shows RRDE experiments for polycrystalline gold electrodes in (20 % O_2_+80 % Ar)‐saturated 0.1 m Ca(ClO_4_)_2_ containing DMSO. For higher roughness factors, the CV shape is similar to the DEMS case in the previous chapter; the ORR starts around −0.8 V in cathodic direction with peroxide formation. However, in case of the lowest electrode roughness (Figure 7a), the decreasing reduction current indicates a transition from peroxide to superoxide formation. This transition from the 2 e^−^ reduction to a 1 e^−^ reduction had been found before and explained by the formation of a CaO/CaO_2_ adsorbate layer, which had also been characterized by XPS.^[29]^ The ORR is accompanied by an increase in ring current, which is astonishingly low as long as peroxide is the main product. This is visible in Figure 7b as shoulder (ca. −1.2 V) in the ring current and in Figure 7e,h as plateau during ORR.

The ring current due to $O_{2}^{-}$ reoxidation at the lower potential limit corresponds to that expected from the disc current. The ring current caused by re‐oxidation of the generated peroxide does not increase with rotation frequency but decreases with time. This will be addressed further below. During the anodic scan only a small current for the OER is observed at the disk electrode visible in Figure 7c,f,i. With increasing electrode roughness, the overall current during the anodic scan increases, especially below 0 V. In Figure 7c,f,I, the anodic peak above 0 V increases with increasing rotation frequency, whereas in Figure 7i the current below 0 V decreases with increasing rotation frequency (cf. remark B in the Supporting Information).

**Figure 8:**
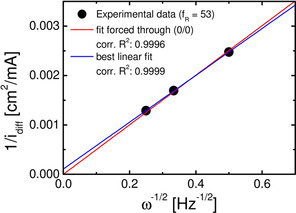
Plot of the inverse diffusion‐limited current (at −1.25 V for f_R_=53; Figure 7d) vs the inverse square root of the rotation frequency (dots), linear fit forced through (0/0) (red line) and best linear fit (blue line) confirming diffusion limitation.

Figure 8 shows a plot of inverse limited ORR current vs inverse square root of rotation frequency taken from Figure 7d. The plot shows that the ORR at electrodes with *f*_R_≥53 really is diffusion limited, as the fit deviates very little from a fit going through the origin. Using the dynamic viscosity of DMSO^[37]^ a diffusion coefficient of 2.48×10^−5^cm^2^ s^−1^ is obtained from the slope. This value is close to those obtained by a non‐electrochemical method for 0.1 m solutions of Li^+^, Na^+^, K^+^, Rb^+^, Cs^+^ and TBA^+^ in DMSO.[^38^, ^39^] Here, we used this method, in which the rate of diffusion of a volatile species through a thin, liquid layer between two membranes is determined by mass spectrometric detection for our 0.1 m Ca^2+^‐containing DMSO. We obtained $D_{O_{2}}$ =2.18×10^−5^cm^2^ s^−1^, which is in good agreement with the value obtained from electrochemical RDE measurements.

Concerning the decrease of peroxide formation rate during ORR at smooth electrodes and transition to the 1 electron superoxide formation, we had suggested that the electrode is slowly covered by an adsorbate (CaO_2_ or CaO), which not only completely blocks peroxide formation, but also inhibits superoxide formation to some extent as it becomes a thin, closed layer.^[29]^ To confirm this we conducted an RRDE experiment, where the Au electrode was first saturated with this CaO_2_/CaO adsorbate by holding the potential at −1.6 V during the scan for 5 min at 9 Hz rotation frequency. As the CaO_2_/CaO layer forms at this potential, superoxide formation becomes predominant as visual by the current decreasing to a constant value of −265 μA cm^−2^ (i. e., 50 % of the initial current). To exclude a thick layer of CaO_2_ deposit as being the reason for the inhibiting effects, the disk potential was then stepped to −0.75 V (just before ORR region) to dissolve possibly formed multilayers for 10 min (still at 9 Hz rotation frequency). After this procedure the Au surface is only covered with a CaO_2_/CaO adsorbate layer (as the thick deposit is dissolved) and CVs were recorded starting in cathodic direction, consecutively opening the anodic limit from −0.75 V to +0.7 V (see Figure S2).

**Figure 9:**
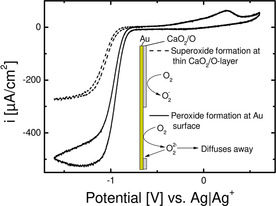
Voltammetry of a CaO_2_/O covered (dashed line) and regenerated (solid line) Au (exchange disk) electrode in (20 % O_2_+80 % Ar)‐saturated 0.1 m Ca(ClO_4_)_2_ containing DMSO at 20 mV s^−1^ and 9 Hz rotation frequency vs Ag/Ag^+^ using a usual H‐cell. The disk current is normalized o the geometrical disk surface area. The disk electrode roughness was f_R_≈13.

Figure 9 is an extract of Figure S2, showing the CV after the Au electrode is covered with this thin, but closed CaO_2_/CaO film starting from the hold potential of −0.75 V in cathodic direction (dashed line) and a CV (solid line) after the electrode is regenerated during the consecutively opening of the upper potential limit to +0.7 V. (Simply cycling to positive potentials (above 0.3 V) to desorb the adsorbate layer also would have been sufficient). But the approach used here (Figure S2) further shows that the electrode begins to slowly reactivate as the upper limit reaches 0.3 V, which indicates 0.3 V as the potential required for CaO_2_/CaO adsorbate oxidation. Note that in the first sweeps up to 0.4 V oxidation of this adsorbate is slow and remains incomplete, whereas in subsequent sweeps the maximum current is at 0.25 V (peak potential). This indicates a nucleation and growth behavior for the free sites, as observed earlier for Ca^2+^ electrolytes on smooth Au electrodes^[29]^ and for Na^+^ electrolytes on smooth Pt (but not on Au).^[40]^

As shown schematically in Figure 9, on top of the CaO_2_/CaO film oxygen is exclusively reduced to superoxide. The continuous reduction is astonishing, as a layer of Li_2_O_2_ almost completely inhibits further ORR on Au and Pt electrodes.^[28]^ As the electrode is regenerated by oxidative stripping, so that oxygen has access to the gold surface, peroxide is formed as predominant product, thus confirming our suggestion. The ring current shown in Figure S2 confirms superoxide (being completely re‐oxidized) or peroxide (being re‐oxidized to a small extent) as the main product. A very similar approach for smooth gold electrodes showed a stronger suppression of superoxide formation (Figure S2 and Ref. [29]). This is explained by the influence of the rough surface leading to a more defective CaO_2_/CaO adsorbate.

The decrease of peroxide formation and transition to superoxide formation is not observed in the DEMS measurements (Figure 3[^21^, ^22^]). This is due to lower mass transport in the DEMS cell, while the large hysteresis concerning ORR for smooth electrodes in the DEMS cell already is an indication for the formation of the CaO_2_/CaO film, as it is caused by the decrease in electrode activity. Therefore, we assume that the adsorbate layer formation is a slow reaction competitive to the formation of soluble peroxide. This experiment and the corresponding experiment on smooth gold^[29]^ identify the adsorbate as reason for inhibition of peroxide formation, which persists after slow dissolution of the thicker CaO_2_ bulk layer formed during ORR.

The normalized ring current during peroxide oxidation at the disk electrode (Figure 7b,e,h) is much smaller than expected: it should be larger (oxidation of peroxide to oxygen) or at least as large (oxidation of peroxide to superoxide) as the ring current during superoxide formation. In the following we will show that this is due to sluggish oxidation kinetics by increasing the effective rate of peroxide oxidation at the ring electrode.

**Figure 10:**
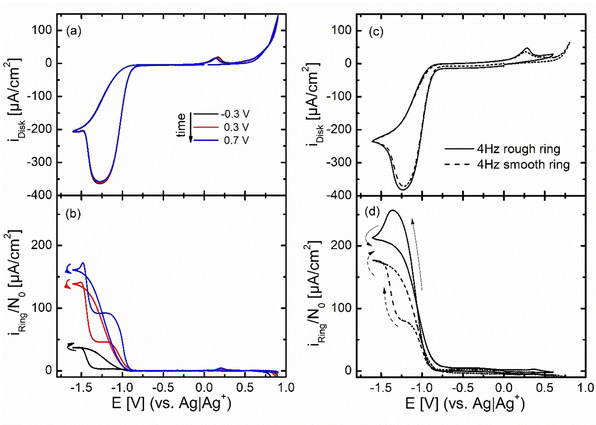
a,b) Cyclic voltammetry of gold (thin gap disk electrode) in (20 % O_2_+80 % Ar)‐saturated 0.1 m Ca(ClO_4_)_2_ containing DMSO at 20 mV s^−1^ and 4 Hz rotation frequency vs Ag/Ag^+^ in a usual H‐Cell. The disk current (a) is normalized to the geometrical electrode surface area and the ring current (b) is normalized to the theoretical collection efficiency (*N*_0_) and the geometrical WE surface area. The ring potential was varied as indicated by the color code. c,d) Cyclic voltammetry of gold (exchange disk electrode) in (20 % O_2_+80 % Ar)‐saturated 0.1 m Ca(ClO_4_)_2_ containing DMSO at 20 mV s^−1^ and 4 Hz rotation frequency vs Ag/Ag^+^ in a usual H‐Cell. The disk current (c) is normalized to the geometrical electrode surface area and the ring current (d) is normalized to the theoretical collection efficiency (*N*_0_) and the geometrical WE surface area. The ring potential was 0.4 V. The electrode roughness of the disk electrodes in (c) were *f*_R_≈3 for both, while the ring electrode was polished (dashed line) or roughened (solid line). The arrows (b,d) indicate the sweep direction. Data is smoothed by using the Savitzky‐Golay method with 25 points of window.

In the first experiment (Figure 10a,b) the ring potential was increased from −0.3 V to +0.7 V. All ring currents increase with increasing ring potential, which indicates kinetic limitations. As already shown in Ref. [21] a minimum potential of 0.3 V is required to achieve oxidation of the superoxide at low potentials; higher ring potentials only lead to slightly more efficient re‐oxidation. The necessity for such a high ring potential to achieve oxidation of superoxide which should be oxidized around −0.3 V was explained by the formation of a contact ion pair between Ca^2+^ and $O_{2}^{-}$ . Anyway, superoxide oxidation is sufficiently fast at a ring potential of 0.3 V (or 0.4 V as used in the other RRDE measurements). However, oxidation of peroxide is not complete even at 0.7 V (note that the normalized ring current should ideally be the same as the disk current). This indicates a very sluggish peroxide oxidation kinetics; increasing the ring potential further would lead to stronger DMSO decomposition/gold dissolution and therefore is not an option. We used another approach in the second experiment (Figure 10c,d). Here the ring electrode was roughened using the same method as for polycrystalline gold disk electrodes leading to a ring electrode roughness of *f*_R_≈32. (The dependence on rotation rate is shown in Figure S3). Thus, the amount of detected peroxide is strongly increased: In Figure 10 the ring current now even resembles the shape of the ORR at the disk. This effect of kinetic limitation also explains the independence of the ring current on the rotation frequency (Figure 7e,h), while the deactivation of the ring electrode with time can be explained by slow accumulation of decomposition products (e. g., from DMSO decomposition by singlet‐oxygen).[^25^, ^26^, ^27^]

Qualitatively it is already obvious from the size of the peroxide formation current, which is much larger than the oxidation peak at the disc at 0.3 V that peroxide does not accumulate at the disc electrode (at least under convection) but rather dissolves. To confirm this more quantitatively, and to separate the superoxide and peroxide contributions, we use Figure 7 for *f*_R_=3 and introduce the superoxide share *χ*. Using the approach shown in the supporting information for the experiment in Figure 7a–c, the obtained charge densities (referring to the geometrical surface area) are 6987 μC cm^−2^ (4 Hz), 7998 μC cm^−2^ (9 Hz) and 8332 μC cm^−2^ (16 Hz; this only provides a rough estimate of the peroxide contribution). Comparing these values with the peroxide oxidation charge densities at the disk (evaluated from the anodic and cathodic scan prior to the steep increase in disk current close to the upper potential limit) – 1045 μC cm^−2^ (4 Hz), 1215 μC cm^−2^ (9 Hz) and 1338 μC cm^−2^ (16 Hz) – it is evident that most of the peroxide that is formed while the adsorbate layer is incomplete is dissolving. The discrepancy between peroxide formation and oxidation charge is even much larger as exclusively peroxide is formed on roughened electrodes. The OER charge on smooth polycrystalline Au electrodes without convection is larger than the values under convection, owing to the contribution of undissolved bulk CaO_2_ to OER, which is mostly dissolved under convection.

In Figure 5 and Figure 7 one observes a positive shift of ORR half‐wave potential with increasing electrode roughness. In the DEMS measurement, the shift in half‐wave potential will not be quantified, as the whole electrode was roughened in a hanging meniscus setup, while only the inner part of the electrode surface has contact to the electrolyte in the DEMS cell. Therefore, only the RRDE experiment is used for further evaluation to avoid uncertainties in electrode roughness. To quantify the half‐wave potential shift, we start with the current density, which is given by the Koutecký‐Levich equation [Equation 1]:

(1) $$\frac{1}{i}=\frac{1}{i_{D}}+\frac{1}{i_{K}}$$

with the diffusion‐limited current density $i_{D}$ and kinetic current density $i_{K}$ . For a given current density i being a fraction 1/x of the diffusion‐limited current $i_{D}$ such as i /2 at the half‐wave potential, Equation (1) gives Equation 2:

(2) $$\frac{x}{i_{D}}=\frac{1}{i_{D}}+\frac{1}{i_{K}}$$

After rearranging Equation (2) and inserting the expression for $i_{K}$ , this leads to Equation 3:

(3) $$zF\left[k_{0}f_{R}\right]e^{-\frac{\alpha_{app}FE}{RT}}=\;\frac{i_{D}}{x-1}=b$$

where $k_{0}$ is the rate constant per unit true surface area, $f_{R}$ is the roughness factor and $\alpha_{app}$ is the apparent cathodic transfer coefficient. Converting the expression leads to Equation 4:

(4) $$E=\;\frac{RTln\left(f_{R}\right)}{\alpha_{app}F}+\frac{RTln\left(\frac{zFk_{0}}{b}\right)}{\alpha_{app}F}$$

As $i_{D}$ is independent of E and $f_{R}$ , this gives the proportionality between the roughness factor and the potential for a constant current density i , such as the current at the half‐wave potential [x=2 ; Eq. 5]:

(5) $$E_{\frac{1}{2}}=\frac{RT}{\alpha_{app}F}ln\left(f_{R}\right)+constants$$

The apparent transfer coefficient ($\alpha_{app}$ ) can thus be obtained from the slope of $E_{\frac{1}{x}}$ vs. $ln\left(f_{R}\right).$

**Figure 11:**
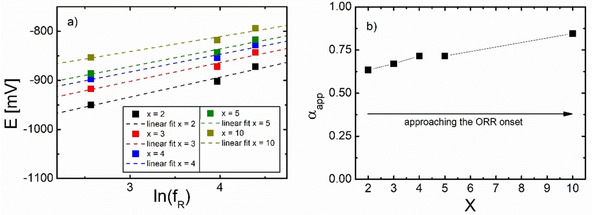
a) Plot of *E*_1/*x*_ vs. ln(*f*_R_) where squares show *E*_1/*x*_ for *i*=*i*_D_/x and dashed lines show linear fit. b) Plot of $\alpha_{app}$ vs. *x* (black dots). The arrow indicates approaching the ORR onset and thus the more kinetically controlled region. Measurements were conducted in 0.1 m Ca(ClO_4_)_2_ containing DMSO in an RRDE setup at 9 Hz rotation frequency and 20 mV s^−1^ sweep rate.

Figure 11a shows the plot of the logarithm of the roughness factor vs the ORR potential for i being a fraction 1/x of $i_{D}$ . From the slopes, one obtains reasonable values for the apparent transfer coefficient $\alpha_{app}$ (Figure 11b). Figure 11b further shows that approaching the ORR onset, $\alpha_{app}$ approaches the value of 0.9, which indicates an initial, equilibrated 1 $e^{-}$ step, followed by a slow process (e. g., slow formation of the ${CaO}_{2}^{+}$ ion‐pair). This is in agreement with the observed Tafel slope of close to 60 mV dec^−1^ for rough electrodes (*f*_R_=53 and 81; see Figure S4. At larger overpotentials (or *X*=2) $\alpha_{app}$ approaches 0.5 in agreement with the Tafel plot yielding a slope of 120 mV Figure S4. This could indicate a transition to a slow 1^st^ electron transfer caused by partial blocking of the surface, in particular of active sites, in the course of the potential sweep. Moreover, chemical dissolution of the peroxide formed after the 2^nd^ e‐transfer might be too slow at large overall rates, causing such a blocking only at larger rates and thus higher overpotentials. A preliminary Tafel analysis for electrodes with low *f*_R_ yields Tafel slopes of 90 mV already during the ORR onset caused by the same reason.

## Discussion

**Figure 12:**
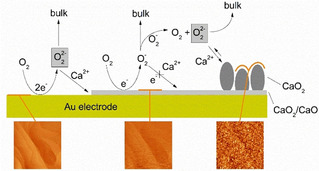
ORR/OER mechanism in Ca^2+^‐containing DMSO on Au electrodes.

Collecting the results in this study, we are now able to suggest a consistent mechanism for the ORR mechanism in Ca^2+^‐containing DMSO on gold and how it changes with electrode roughness (Figure 12).

Regarding the voltammetry we will have a closer look at the ORR and OER separately and define the ORR region from −0.8 V to −1.6 V and the OER region from −0.5 V to 1.0 V vs Ag/Ag^+^ respectively.

### ORR

The predominant product during ORR is slightly soluble peroxide formed via an initial 1 $e^{-}$ reduction in equilibrium followed at least by a further 1 $e^{-}$ reduction step, until a complete adsorbate layer of CaO_2_/CaO is formed. Thereafter, superoxide is formed.

- It was already shown in earlier studies and confirmed in this study, that a two electron process accompanied with oxygen consumption is observed below −0.8 V in the investigated system.[^21^, ^22^]
- The formed peroxide is only slightly soluble: In the absence of convection, it precipitates in a nucleation and growth mechanism at the surface, predominantly at defects; it can slowly dissolve as demonstrated in the AFM experiments, for example. Under convection (which is more severe in the RRDE experiments than in the DEMS experiments) it is completely dissolved and transported to the ring electrode. There, its oxidation is slow and requires high potentials even at rough ring electrodes.
- Peroxide formation is initiated by a one electron process (most likely superoxide formation) as indicated by the Tafel slope obtained from the current voltage curves and – indirectly – from the shift of the half‐wave potential of ORR with electrode roughness.
- In immediate contact with the Au surface is an adsorbed layer of CaO_2_ or CaO,^[29]^ which is also stable after dissolution of bulk CaO_2_ and which inhibits further reduction of O_2_ to peroxide and therefore leads to the observed change in electrode activity (hysteresis) and transition from peroxide formation to superoxide formation. This assumption of an adsorbate blocking further peroxide formation stems from the small oxidation charge (typically below the charge of 390 μC cm^−2^ (real surface area) corresponding to a monolayer) observed for oxidative stripping of the adsorbate in subsequent anodic sweeps at the RRDE and AFM after waiting for complete dissolution.
- After completion of the adsorbate layer soluble superoxide is the predominant product during ORR. At smooth Au, also superoxide formation is blocked 300 s after potential stop at −1.6 V vs Ag/Ag^+^, while on rough electrodes, the current for superoxide formation is larger and limited by diffusion without notable decay within 150 s of observation after completion of the adsorbate layer.
- The transition of peroxide to superoxide formation is indicated by an increasing ring current in RRDE generator collector experiments because peroxide oxidation at the ring electrode is limited by kinetics and not by diffusion (superoxide has faster oxidation kinetics).
- On smooth disk electrodes in RRDE experiments, the superoxide formation is also partially hindered by the CaO_2_/CaO‐adsorbate layer,^[29]^ while on rough electrodes this is not the case, indicating a more defective adsorbate and thus more facile conduction.
- The electrochemically formed, soluble peroxide and the peroxide possibly formed via disproportionation deposits on the electrode surface as particles, if no convection is present. The particle formation is clearly visible in AFM measurements without convection. So far it is unclear, if the deposit originates from soluble peroxide formed prior completion of the adsorbate layer, from peroxide formed via superoxide disproportionation or both.

### OER

The CaO_2_/CaO‐adsorbate oxidation starts at potentials from −0.5 V to 0.3 V depending on the degree of adsorbate layer completion.

- At smooth electrodes the adsorbate layer is formed during one cycle under the RRDE measurement conditions in this study, while the anodic peak associated with adsorbate layer oxidation is located around 0.3 V. Moreover, for *f*_R_=13, after completion of the adsorbate layer, regeneration of the electrode by oxidative stripping of the adsorbate, starts at 0.3 V. For very rough electrodes, an anodic current can be observed starting from −0.5 V, which indicates easier oxidation of the incomplete adsorbate layer, as the layer is not finished during one cycle (indicated by diffusion‐limited peroxide formation during the ORR on rough electrodes).
- Oxidation of the adsorbate layer and of thicker peroxide layers occur at similar potentials.
- We had shown before, that oxidation of the superoxide CaO_2_^+^ (at the ring electrode after formation at the disc) requires overpotentials of more than 0.5 V. This had been ascribed to the stabilization of the superoxide by forming a contact ion pair with Ca^2+^. This effect is much more drastic for peroxide oxidation, which requires overpotentials of 1.5 V at a smooth ring electrode. This is not really astonishing because the reversible thermodynamic potential of CaO_2_/O_2_ is about 1.5 V more positive than that of the superoxide Ca(O_2_)_2_/O_2_.^[29]^ Since superoxide formation can occur only around −1 V (vs. Ag/Ag^+^), and since peroxide formation involves superoxide as an intermediate, it is the reduction to peroxide which requires a large (negative) overpotential, whereas its reoxidation occurs close to the thermodynamic potential. The question remains whether a catalyst or another solvent could be found for a possible calcium/oxygen battery allowing the direct 2‐electron reduction to peroxide at a more positive potential than to superoxide in Ca containing electrolytes.

## Conclusions

In this work, we investigated the influence of atomic surface structure and electrode roughness on the ORR in Ca^2+^‐containing DMSO and thus shed some light on the mechanism of oxygen reduction on gold surfaces. As we have shown, the atomic surface structure has a minor influence on the ORR in presence of convection. We found peroxide as main product during ORR both on single crystalline and polycrystalline gold surfaces as indicated by the number of transferred electrons in DEMS measurements under convection. Concerning the influence of electrode roughness, we found that a rough gold surface allows for continuous peroxide generation under the typical measurement conditions, whereas at smooth electrodes it is soon replaced by superoxide formation. However, under longer ORR without intermediate OER (i. e., oxidative stripping of the blocking adsorbate) peroxide formation is not maintained neither on the rough electrodes; instead, diffusion‐limited superoxide formation is observed, whereas at the smooth electrode the OER is completely suppressed after some time.

The reason for this behavior is the formation of a CaO_2_ or CaO adsorbate on gold electrodes that inhibits further peroxide formation during ORR as soon as it forms a closed layer; instead, superoxide is formed at defects of this layer. Its formation requires more time at rough electrodes because it competes with peroxide formation and is diffusion limited. (For a roughness of 50, its formation would thus take 50 times as long as for a roughness of 1.) At Au single crystal electrodes, on the other hand, its formation is much slower probably because nucleation sites are missing. At smooth electrodes, this adsorbate layer ultimately is sufficiently defect – free to also inhibit superoxide formation, whereas at rough electrodes such a poisoning could not be observed. On Pt electrodes, the behavior is somewhat different, as already shown in Ref. [21, 22]. This will be examined in more detail in a forthcoming paper.

The soluble peroxide and superoxide can be oxidized at a collector electrode in a generator collector arrangement (e. g., RRDE), but the kinetics of the reoxidation is slow for superoxide (due to stabilization in an ion pair with Ca^2+^) and even slower for the peroxide. In the absence of convection, the formed peroxide (either via the 2^nd^ electron transfer at the electrode surface or possibly via disproportionation of the superoxide in solution) further deposits as CaO_2_ particles on top of the CaO_2_/CaO adsorbate layer as indicated by AFM measurements in this study; these experiments also demonstrated that this peroxide can be dissolved.

Furthermore, we found in DEMS and RRDE measurements a dependency of the half‐wave potential on electrode roughness as predicted by theory and showed a further RRDE approach to obtain reasonable values for the apparent transfer coefficient ($\alpha_{app}$ ). Using this approach combined with the Tafel slopes we conclude an initial, equilibrated one electron process followed by a slow process (e. g., slow formation of the ${CaO}_{2}^{+}$ ion pair). This proves the electrode roughness to be a valuable tool for investigations on kinetics.

## Conflict of interest

The authors declare no conflict of interest.

## Supporting information

As a service to our authors and readers, this journal provides supporting information supplied by the authors. Such materials are peer reviewed and may be re‐organized for online delivery, but are not copy‐edited or typeset. Technical support issues arising from supporting information (other than missing files) should be addressed to the authors.

Supplementary
