# Supplementary material for: Electrochemical Reduction of O2 in Ca2+‐Containing DMSO: Role of Roughness and Single Crystal Structure
Source: ChemSusChem. 2021 May 13;14(12):2564–75. doi: 10.1002/cssc.202100364 (PMC8252535; doi:10.1002/cssc.202100364)
Supplement: Supplementary file 1 — Supplementary [file CSSC-14-2564-s001.pdf]

ChemSusChem

Supporting Information

Electrochemical Reduction of O_2 in Ca^{2+}-Containing DMSO: Role of Roughness and Single Crystal Structure

Andreas Köllisch-Mirbach, Inhee Park, Martina Hegemann, Elke Thome, and Helmut Baltruschat*© 2021 The Authors. ChemSusChem published by Wiley-VCH GmbH. This is an open access article under the terms of the Creative Commons Attribution License, which permits use, distribution and reproduction in any medium, provided the original work is properly cited.

1. Calculation of the roughness factor:

f_R denotes the electrodes roughness factor and is given by

$$f_\mathrm{R} = \frac{A_\mathrm{real}}{A_\mathrm{geo}} \tag{S1}$$

With A_real as real surface area and A_geo as geometrical surface area. As the current of an electrode reaction is proportional to A_real, one directly obtains f_R via the ratio of the real charge Q_real and the geometrical charge Q_geo

$$f_\mathrm{R} = \frac{Q_\mathrm{real}}{Q_\mathrm{geo}} \tag{S2}$$

While Q_geo is available from the theoretical number of atoms on the surface or an empirical value for a given potential interval.

1.1. Gold electrodes:

For polycrystalline gold electrodes an empirical value (390 µC/cm^2) for Q_geo concerning the oxygen region in 0.5 M H_2SO_4 is known and well accepted as the charge density for a monolayer.[1] Thus integration in the relevant oxygen region via

$$Q_\mathrm{real} = \int I v_\mathrm{s}^{-1} dE \tag{S3}$$

With v_s as sweeprate given by

$$v_\mathrm{s} = \frac{dE}{dt} \tag{S4}$$

Gives Q_real and further division by 390 µC/cm^2 yields f_R.

2. Calculation of the number of transferred electrons in DEMS measurements:

The potential dependent number of transferred electrons z is obtained from DEMS measurements by correlating the faradaic current I_F at the electrode with the ion current I_ion of the mass spectrometer. Faradaic and ion current are proportional, with K^* as the constant of proportionality. The ion current is given by

$$I_{\mathrm{ion}} = K^* \frac{I_{\mathrm{F}}}{z} \tag{S5}$$

Leading to

$$z = K^* \frac{I_{\mathrm{F}}}{I_{\mathrm{ion}}} \tag{S6}$$

For the number of transferred electrons, while K^* is obtained by calibration via a well-known reaction. In this case the ORR in tetrabutylammoniumperchlorate ($TBAClO_4$) containing DMSO, where only superoxide is formed ($z = 1$).[2-4] Thus K^* is given by

$$K^* = \frac{I_{\mathrm{ion}}}{I_{\mathrm{F}}} \tag{S7}$$

3. Separation of superoxide and peroxide contribution:

Assuming eq.1-3 to hold, χ can be derived from the disk current I_{disk} and ring current I_{ring} via:

$$I_{\mathrm{disk}} = 2F \frac{dn_{\mathrm{O_2}}}{dt} - F \frac{dn_{\mathrm{O_2}}}{dt} * \chi \tag{1}$$

$$I_{\mathrm{ring}} = 2FN_{\mathrm{P}} \frac{dn_{\mathrm{O_2}}}{dt} * (1 - \chi) + FN_{\mathrm{S}} \frac{dn_{\mathrm{O_2}}}{dt} * \chi \tag{2}$$

With N_{P} and N_{S} as collection efficiency of peroxide and superoxide. After converting the expression to

$$\frac{dn_{\mathrm{O_2}}}{dt} = \frac{I_{\mathrm{disk}}}{F(2 - \chi)} = \frac{-I_{\mathrm{ring}}}{F(2N_{\mathrm{P}} + \chi N_{\mathrm{S}} - 2\chi N_{\mathrm{P}})} \tag{3}$$

One obtains the superoxide share

$$\chi = \frac{2(I_{\mathrm{ring}} + N_{\mathrm{P}} I_{\mathrm{disk}})}{I_{\mathrm{ring}} + 2N_{\mathrm{P}} I_{\mathrm{disk}} - N_{\mathrm{S}} I_{\mathrm{disk}}} \tag{4}$$

To further obtain N_P and N_S via

$$N_{\mathrm{P}} = \frac{I_{\mathrm{P}}^{\mathrm{ring}}}{I_{\mathrm{P}}^{\mathrm{disk}}} \tag{5}$$

$$N_{\mathrm{S}} = \frac{I_{\mathrm{S}}^{\mathrm{ring}}}{I_{\mathrm{S}}^{\mathrm{disk}}} \tag{6}$$

Were $I_{\mathrm{S}}^{\mathrm{disk}}$, $I_{\mathrm{S}}^{\mathrm{ring}}$, $I_{\mathrm{P}}^{\mathrm{disk}}$ and $I_{\mathrm{P}}^{\mathrm{ring}}$ are taken from the voltammogram as shown in Figure S5 assuming that $I_{\mathrm{S}}^{\mathrm{disk}}$, $I_{\mathrm{S}}^{\mathrm{ring}}$, $I_{\mathrm{P}}^{\mathrm{disk}}$ and $I_{\mathrm{P}}^{\mathrm{ring}}$ exclusively consist of superoxide/peroxide generation/oxidation respectively. The product of $I_{\mathrm{disk}}(E)$ and $(1 - \chi(E))$ gives the current contribution of peroxide.

Integration of the peroxide formation current leads to the peroxide charge of 6987 μC/cm^2 (4 Hz), 7998 μC/cm^2 (9 Hz) and 8332 μC/cm^2 (16Hz).

4. Calculation of the theoretical peak current density:

Theoretical peak current densities (i_p) were calculated using the Randles-Sevcik equation for a reversible process:

$$i_\mathrm{p} = 0.4463\, C\, z^{3/2}\, F^{3/2}\, (vD/RT)^{1/2} \tag{7}$$

And a totally irreversible process:

$$i_\mathrm{p} = 2.99 * 10^5 * zC\, (\alpha z_\alpha\, Dv)^{1/2} \tag{8}$$

With C as bulk concentration of oxygen, z as number of transferred electrons, z_α as number of transferred electrons during the rate determining step, α as transfer coefficient, F as Faraday constant, v as sweep rate, D as diffusion coefficient of oxygen, R as gas constant and T as temperature. The bulk concentration of oxygen (0.4 mM at 20% O_2) in our electrolyte was determined like in [5, 6].

5. Further remarks:

SI A: Note that the voltammograms of the single crystalline electrodes were not stable upon continuous cycling. The ORR current decreased and the OER current increased with time, thus indicating, that crystallization of CaO_2 becomes more facile over time, which further indicates incomplete oxidation or changes in the surface structure.

SI B: The behavior of the anodic disk current in Figure 7 further indicates the formation of a thin layer. Starting with the rough electrode (see Figure 7(i)), one observes a current below 0 V, which decreases with increasing rotation frequency/time, while the current above 0 V increases with rotation frequency/time. In the picture of an adsorbate that slowly forms a closed layer that absolutely makes sense, as with increasing rotation frequency more peroxide is generated and thus adsorbed on the electrode surface. So as one increases the rotation frequency, the layer becomes more complete, thus increasing the potential required for the oxidation of the adsorbate. For lower electrode roughness (see Figure 7(c, f)) this transition is not observed, but the anodic peak current shifts to more positive potentials leading to the same conclusion.

6. Additional Figures:

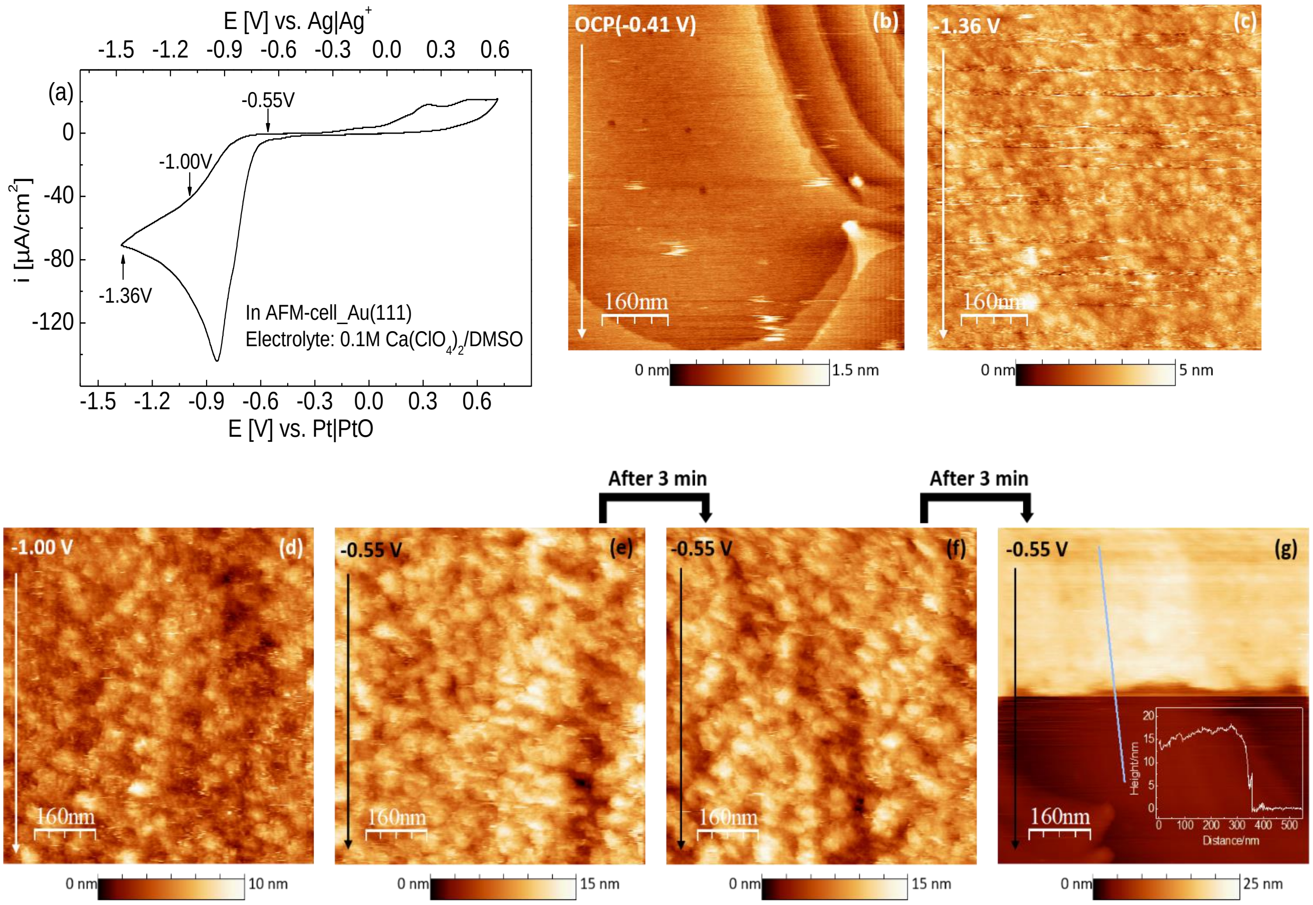

Figure S1 (a) CV on Au(111) in AFM-cell at 5 mV/s during AFM measurement. In situ AFM images showing the topography on Au(111) at OCP (b), -1.36 V vs. Pt|PtO (c), -1.00 V vs. Pt|PtO (d), -0.55 V vs. Pt|PtO (e)-(g). The inset image of (g) shows the height profile. To avoid the tip-induced influence on the electrochemical reaction we lifted up the AFM tip by 100 µm during ORR process and then re-approached it to the electrode surface after the ORR reaction. The arrow in the image represents the scan direction. A set point of AFM was 4nN, bias was 50 mV Integral and proportional gains were 8 and 9, respectively. Scan size was 800X800 nm² and scan rate was 3.08 nm/s.

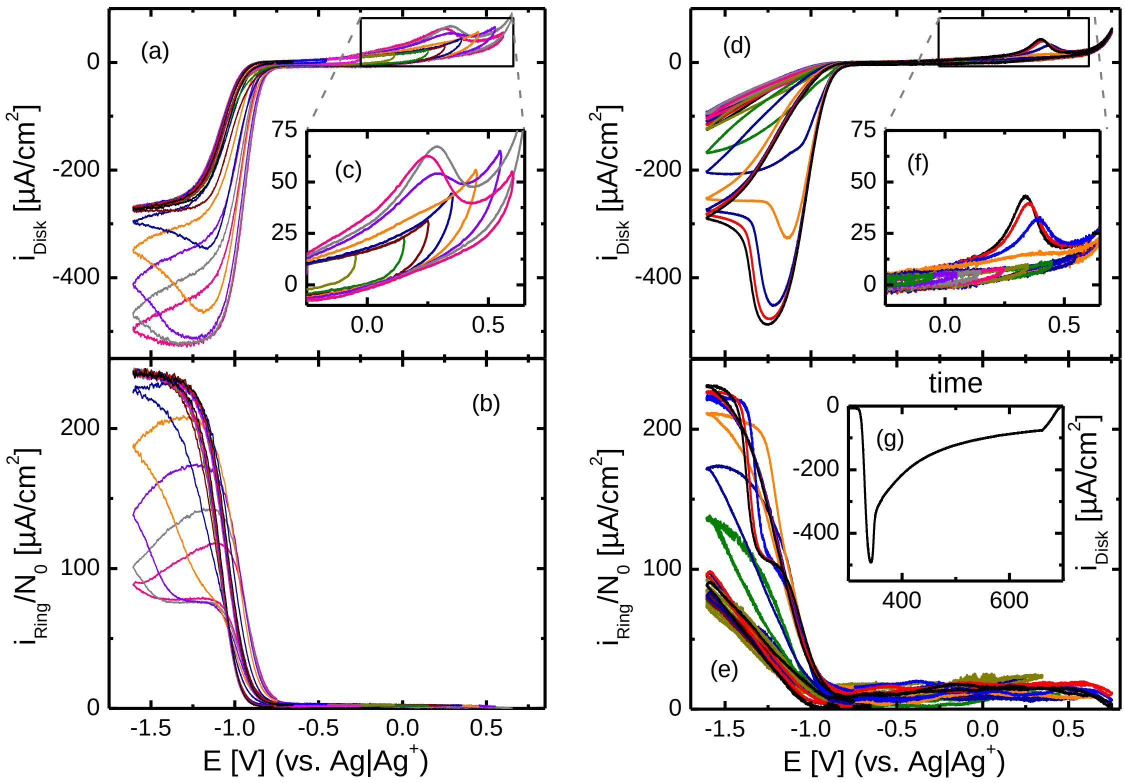

Figure S2 Cyclic voltammetry of roughened gold (left, at f_R = 13) and smooth gold (right, at f_R = 3, data from [7]) in (20% O_2 + 80% Ar)-saturated 0.1 M $Ca(ClO_4)_2$ containing DMSO at 20 mV/s, E_{ring} = 0.4 V and 9 Hz rotation frequency vs Ag|Ag+ using a usual H-cell. Shown CVs are recorded after stopping the potential at -1.6 V for 5 min and jumping to -0.75 V for 10 min afterwards. The upper limit in this experiment is opened consecutively after each cycle until the disk electrode was regenerated by oxidative stripping of the CaO_2/CaO-adsorbate. Disk current density is shown in (a, d). The ring current (b, e) is normalized to the collection efficiency N_0 and the geometrical surface area of the disk electrode for 1 to 1 comparability. The anodic signal is magnified in (c, f). (g): The disk current transient at smooth Au during the 300 sec potential stop at -1.6V vs. Ag|Ag+.

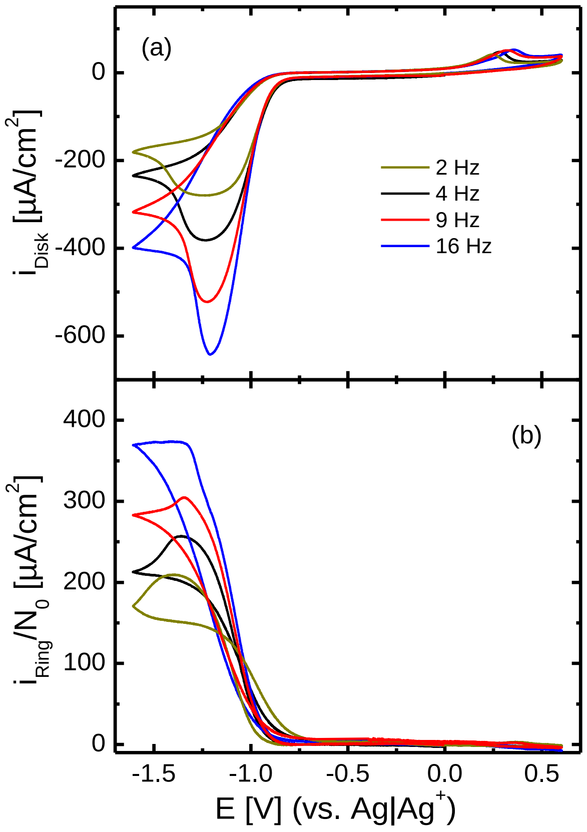

Figure S3 Cyclic voltammetry of gold (exchange disk electrode with $f_R \approx 3$) in (20% O_2 + 80% Ar)-saturated 0.1 M $Ca(ClO_4)_2$ containing DMSO at 20 mV/s, E_{ring} = 0.4 V and 9 Hz rotation frequency vs Ag|Ag$^+$ using a usual H-cell. Disk current density is shown in (a). The ring current (b) is normalized to the collection efficiency N_0 and the geometrical surface area of the disk electrode for 1 to 1 comparability. The ring electrode in this experiment is roughened leading to $f_R \approx 32$.

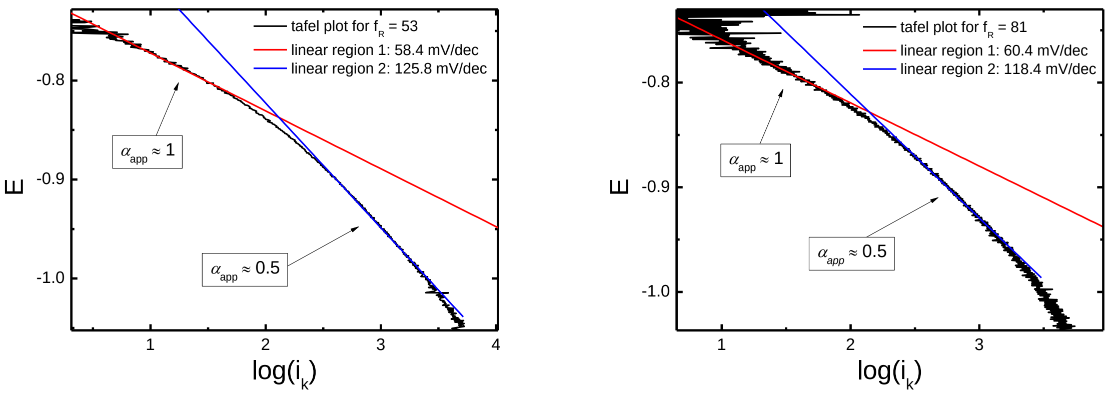

Figure S4 Tafel plot for f_R = 53 and 81. Tafel analysis refers to Figure 7d+g.

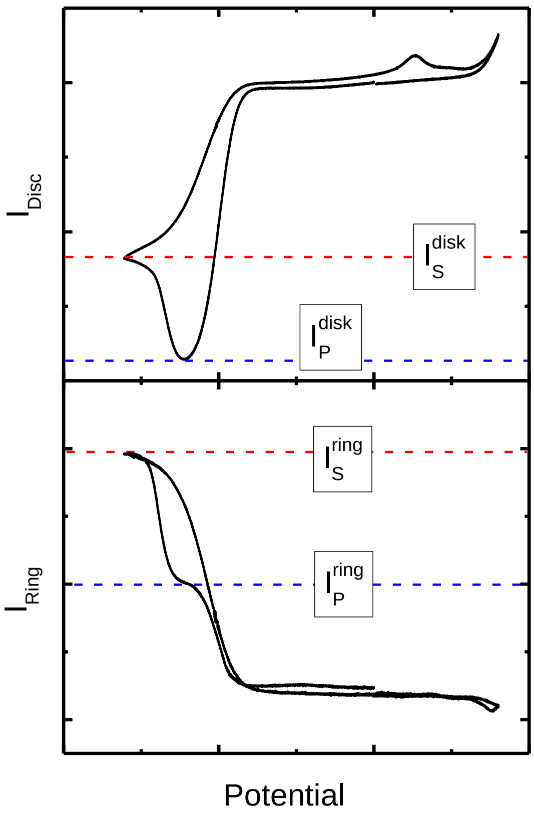

Figure S5 Exemplary determination of I_S^{disk}, I_S^{ring}, I_P^{disk} and I_P^{ring} from the voltammogram.

References

[1] S. Trasatti, O. A. Petrii, *Journal of Electroanalytical Chemistry* **1992**, *327*, 353.

[2] C. O. Laoire, S. Mukerjee, K. M. Abraham, E. J. Plichta, M. A. Hendrickson, *Journal of Physical Chemistry C* **2010**, *114*, 9178.

[3] C. O. Laoire, S. Mukerjee, K. M. Abraham, E. J. Plichta, M. A. Hendrickson, *Journal of Physical Chemistry C* **2009**, *113* 20127.

[4] C. Bondue, P. Reinsberg, A. A. Abd-El-Latif, H. Baltruschat, *Physical Chemistry Chemical Physics* **2015**, DOI: 10.1039/c5cp04356e.

[5] P. H. Reinsberg, P. P. Bawol, E. Thome, H. Baltruschat, *Analytical chemistry* **2018**, *90*, 14150.

[6] P. P. Bawol, P. H. Reinsberg, H. Baltruschat, *Analytical chemistry* **2018**, *90*, 14145.

[7] P. P. Bawol, P. H. Reinsberg, A. Koellisch-Mirbach, C. J. Bondue, H. Baltruschat, *ChemRxiv (Preprint)* **2020**.
